# Recording artist career comparison through audio content analysis

**DOI:** 10.1098/rsos.241647

**Published:** 2025-07-09

**Authors:** Nick Collins

**Affiliations:** ^1^ Music Department, Durham University, Durham, UK

**Keywords:** music information retrieval, audio content analysis, recording artist careers

## Abstract

Audio content analysis can be deployed to examine relationships within and between collected works of different music artists, allowing a new approach to comparative analysis of recorded music within the domain of computational musicology. Although current-generation automatic transcription retains some flaws with respect to expert human analysis, there is a consistency to applying the same algorithms on disparate works, and the benefit of tireless calculation with explicit open bias. In the present study, three successful alternative rock groups, and three ‘control’ artists, all from either the United States or the UK, are compared with respect to their musical careers through their main recorded releases (spanning the years 1983–2021 for the main three and 1957–2000 for the controls). Statistical measures of variation over time, and the diversity of their recorded output, are used to answer research questions on their studio career and the originality of their work. The techniques explored here are immediately pertinent to study other artists outside of this starting point, and we discuss the potential and challenges of such approaches for the musicology of recorded music.

## 1. Introduction

The field of music information retrieval (MIR) [1–4] provides new opportunities to examine music recordings on larger scales than an individual human music analyst can comfortably and reliably undertake, and thereby new insights into music as an evolving cultural activity [5–7]. In the present study, the career of recording artists is examined through audio features derived across their releases, organized by year. Three well-known and inter-related alternative rock bands are the main targets of the study, alongside three contrasting control cases (particularly contrasting in the gender of singer and era of recording) to help broaden testing of the techniques. The study is carried out on a medium scale, so that the musicologist undertaking the research is fully conversant with the audio recordings being studied, and can thereby comment on machine-led discovery from a human listening perspective, though they would find it prohibitive to transcribe by hand all the 573 songs in question.

Given this comparative approach, pertinent research questions are:

(i) are there changes across an artist’s career observable from audio recordings through automated feature extraction? Can particular critical inflection points be observed, or indicators of early or late work?
(ii) relative to comparator artists, what diversity of recorded output is achieved by a given band with respect to particular musical attributes such as complexity of rhythm, harmonic language or size of timbral space explored? and
(iii) are there any commonalities of career, such as a general slowing of tempi or preference for less high-frequency content in textures (for example, less distortion in rock) as musicians age?

Previous work has looked at a broader scale at many popular music recordings over decades [8], often based on the Million Song Data Set [9] of audio excerpts, for example, observing the cultural evolution of music through topic models [10]. Through audio features, increased prevalence of the bass has been noted in more recent recordings [11], and the Loudness War [12] in recording mastering clearly observed ‘peaking’ around 2007 [13] from its origins in 1990s mastering practices (mastering engineers had pressure to compete over the relative playback volume of CDs). Classical music recordings have been analysed for changing tempo preference and concert pitch reference over time [14]; symbolic music data studies have also been carried out taking advantage of human transcription, for example, the greater use of plagal over perfect cadences within a selected corpus of significant chart hits [15].

Although not with an explicit focus on artist careers, researchers have tackled comparative musicological analysis over corpora, showing differences in energy, tempo and emotion score between the United States (US) and UK commercial popular music [16], and rhythmic differences between US popular music from before and after the year 2000 [17]. Within the musicology of recorded music [18], retrospective career analysis of music artists from a comparative perspective through MIR methods has not, to my knowledge, been previously explored. Engineering work on automatic music transcription, covering such tasks as audio chord detection, rhythm analysis and polyphonic timbre, is an ongoing and unfinished area of endeavour [1,19–24]; nonetheless, we do not hold back from examining some consequences with current generation technology, since it is still productive to examine the technical possibilities for musicology. It is understood that as long as methods and code are fully documented—and all open-source code to reproduce the work accompanies this article—it is possible to revisit that analysis should subsequent technology prove higher quality yet.

We proceed to compare six music artists with respect to timbral-rhythmic audio features, and with harmonic language extracted through audio chord detection. Features are plotted against the year of release to explore the career trajectory of artists; clustering of songs between all artists, similarity matrices between artists and between all albums, comparison of preferences in harmonic language and diversity of timbre and rhythm and of harmony, are investigated. The dataset in the present study is first explained in more detail, before analysis in subsequent sections and discussion of the results so obtained and the potential for such techniques in automatic music career analysis.

## 2. The dataset

This section outlines the artists and their recordings appearing in the dataset for this study. Table 1 lists the three primary alternative rock artists involved (all male), and three contrasting control cases, giving their years of activity, the number of songs and releases contributing to the dataset with total durations, and their associated genre of music as a guide, while acknowledging the problematic categorization problems of stylistic labels [25–27]. Table 2 lists the exact album/Extended Play (EP) sources for each artist with release years, and a further breakdown of number of songs and duration per release. The three main artists selected for comparison in this study are the alternative rock outfits R.E.M. [28], Radiohead [29] and Coldplay [30], whose first album releases are staggered by roughly 10 year gaps (1983, 1993 and 2000), and who have acknowledged influence; Michael Stipe has acted as a mentor to Thom Yorke and Radiohead toured with R.E.M.; Coldplay’s use of falsetto vocal directly follows from the precedent of Thom Yorke’s voice. There are a much wider net of influences that all bands can invoke: for example, the falsetto technique can also be traced to Jeff Buckley and Radiohead were also influenced by such artists as Nick Drake, The Smiths, New Order/Joy Division, and Siouxsie and the Banshees. R.E.M. have often acknowledged other Athens Georgia bands such as the B52s, and Coldplay have explicitly sampled Kraftwerk and made clear a range of pop influences (such as Beyoncé) outside of guitar music. All three bands have an interesting openness on instrumentation, with R.E.M.’s use of mandolin and organ, Radiohead’s turn to electronica with *Kid A* (2000) and Coldplay’s dalliances with pop production. The primary recorded documents are the band’s studio albums; R.E.M. disbanded in 2011, Radiohead’s last new album was in 2016, although there are various related projects of band members, including three recent albums in 2022−2024 from the offshoot band The Smile (Thom Yorke and Jonny Greenwood from Radiohead in a trio with drummer Tom Skinner). Coldplay are still actively releasing music; their latest studio album *Moon Music* came out in November 2024 after the present study was submitted to review and is excluded here from the data analysis, given its relative unfamiliarity to the musicologist. The main studio albums and a few key EPs are taken to represent the bands’ careers here, acknowledging that there are a wider set of b-sides, studio out-takes and live recordings that might have been included, at risk of polluting the composition and audio quality; the band’s own selection of what makes a significant body of songs for an EP or album release is taken implicitly here as the best marker of what musical material encapsulates their recording career.^1^ The selection of these three bands, in particular, is because of the network of influence between them, overlapping over some decades with staggered start and end times, the restriction to male-vocal led alternative rock allowing some focus on other aspects of difference between their careers. The current study also follows from a previous report on the songs of Radiohead [31], allowing a larger contextualization of Radiohead’s work without too far a zoom out.

**Table 1.** Artists included in the present study, breaking down the 573 songs considered.

artist	number of releases	number of songs	total duration	years active	genre	band size
R.E.M.	15	184	11 h 53 min 24 s	1983−2011	indie/alternative rock	4
Radiohead	11	114	7 h 54 min 8 s	1993−2016	indie/alternative rock, electronica	5
Coldplay	12	122	8 h 14 min 59 s	1999−2021 (2024, see main text)	indie/alternative rock, pop (2010s)	4
Patsy Cline	5	44	1 h 49 min 42 s	1957−1962	country, pop (1950s)	1
Buddy Holly	4	47	1 h 43 min 18 s	1957−1960	rock and roll/rockabilly	1
Kirsty MacColl	5	62	3 h 49 min 44 s	1981−2000	indie/alternative rock, country, latin	1

**Table 2.** Recordings by each artist included in the present study.

artist	name of recording	abbreviation	year	number of songs	total duration (minutes/seconds)
R.E.M.	*Murmur*	Murmur	1983	12	44 min 13 s
R.E.M.	*Reckoning*	Reckon	1984	10	38 min 15 s
R.E.M.	*Fables Of The Reconstruction*	Fables	1985	14	48 min 56 s
R.E.M.	*Life's Rich Pageant*	Life's	1986	15	43 min 50 s
R.E.M.	*Document*	Docume	1987	11	39 min 52 s
R.E.M.	*Green*	Green	1988	11	41 min 2 s
R.E.M.	*Out Of Time*	Out Of	1991	11	44 min 10 s
R.E.M.	*Automatic For The People*	Automa	1992	12	48 min 54 s
R.E.M.	*Monster*	Monste	1994	12	49 min 17 s
R.E.M.	*New Adventures In Hi-Fi*	New Ad	1996	14	65 min 33 s
R.E.M.	*Up*	Up	1998	14	64 min 33 s
R.E.M.	*Reveal*	Reveal	2001	12	53 min 44 s
R.E.M.	*Around The Sun*	Around	2004	13	55 min 22 s
R.E.M.	*Accelerate*	Accele	2008	11	34 min 40 s
R.E.M.	*Collapse Into Now*	Collap	2011	12	41 min 6 s
Radiohead	*Pablo Honey*	Pablo	1993	12	42 min 11 s
Radiohead	*My Iron Lung EP*	My Iro	1994	7	24 min 6 s
Radiohead	*The Bends*	The Be	1995	11	44 min 2 s
Radiohead	*OK Computer*	OK Com	1997	12	53 min 28 s
Radiohead	*Kid A*	Kid A	2000	10	49 min 58 s
Radiohead	*Amnesiac*	Amnesi	2001	11	43 min 56 s
Radiohead	*Hail To The Thief*	Hail T	2003	14	56 min 37 s
Radiohead	*Disk 2 In Rainbows*	In R 2	2007	8	26 min 51 s
Radiohead	*In Rainbows*	In Rai	2007	10	42 min 44 s
Radiohead	*The King Of Limbs*	The Ki	2011	8	37 min 34 s
Radiohead	*A Moon Shaped Pool*	A Moon	2016	11	52 min 41 s
Coldplay	*Brothers & Sisters EP*	Brothe	1999	3	10 min 56 s
Coldplay	*Parachutes*	Parach	2000	10	41 min 49 s
Coldplay	*A Rush Of Blood To The Head*	A Rush	2002	11	54 min 13 s
Coldplay	*X&Y*	X&Y	2005	13	62 min 36 s
Coldplay	*Prospekt's March EP*	Prospe	2008	8	27 min 32 s
Coldplay	*Viva La Vida Or Death And All His Friends*	Viva L	2008	10	45 min 54 s
Coldplay	*Mylo Xyloto*	Mylo X	2011	14	44 min 13 s
Coldplay	*Ghost Stories*	Ghost	2014	9	42 min 43 s
Coldplay	*A Head Full Of Dreams*	A Head	2015	11	45 min 52 s
Coldplay	*Kaleidoscope EP*	Kaleid	2017	5	24 min 24 s
Coldplay	*Everyday Life*	Everyd	2019	16	52 min 57 s
Coldplay	*Music Of The Spheres*	Music	2021	12	41 min 51 s
Patsy Cline	*Patsy Cline*	Patsy	1957	12	29 min 49 s
Patsy Cline	*Songs By Patsy Cline EP*	Songs	1957	4	10 min 4 s
Patsy Cline	*EP Patsy Cline*	EP Pat	1961	4	9 min 47 s
Patsy Cline	*Showcase*	Show	1961	12	29 min 22 s
Patsy Cline	*Sentimentally Yours*	Sent	1962	12	30 min 40 s
Buddy Holly	*The Chirping Crickets*	The CC	1957	12	26 min 21s
Buddy Holly	*Buddy Holly*	Buddy	1958	12	24 min 27 s
Buddy Holly	*That'll Be The Day*	That	1958	11	25 min 30 s
Buddy Holly	*The Buddy Holly Story Vol. 2* (posthumous)	The B	1960	12	27 min 0 s
Kirsty MacColl	*Desperate Character*	Desp	1981	11	31 min 43 s
Kirsty MacColl	*Kite*	Kite	1989	15	49 min 22 s
Kirsty MacColl	*Electric Landlady*	Elec	1991	12	51 min 50 s
Kirsty MacColl	*Titanic Days*	Titan	1993	11	47 min 29 s
Kirsty MacColl	*Tropical Brainstorm*	Trop	2000	13	49 min 19 s

In order to give additional perspective to the results, we also analyse three control cases. These were picked carefully to present a contrast in the era of popular music, and of gender of singer; they are also marketed as led by solo singers (albeit with varying backing bands). Two do not overlap in release dates at all, namely Buddy Holly [32] and Patsy Cline [33] (both died tragically in plane crashes in 1959 and 1963, respectively). One additional control case is Kirsty MacColl [34], who herself unfortunately died in 2000, but who was releasing albums overlapping with the bands here in the 1980s and 1990s, in a range of styles including country, indie rock and Latin. The choice of these controls rather than innumerable other possibilities is to compare with female voices (albeit there are female guest vocals on a few isolated R.E.M. and Coldplay tracks), and to compare across a wider span of decades into earlier popular music production and instrumentation trends. Note that Radiohead in particular would have been familiar with Kirsty MacColl, if only from her backing vocals on various Smiths songs; R.E.M.’s non-virtuosic guitar sound is on an evolutionary trail from 1950s to 1960s precedents in rock and roll and country music. The use of a restricted corpus allows a human musicologist to be comfortably familiar with all the music under computational analysis, to assist in full interpretation of results. While the Million Song Dataset [9] might be the object for another study over a larger body of artists, it is non-complete for particular artists across their careers, with songs represented by shorter excerpts, and impossible for a human musicologist to listen to sufficiently for any close readings; the audio analysis is also restricted to data supplied with the set, and does not include specific chord detection data as is used herein.

## 3. Audio features over a recording career

Thirty timbral and rhythmic audio features were extracted using the SuperCollider Music Information Retrieval library (SCMIR)^2^ [35] across 573 audio files; table 3 breaks down the features obtained. SuperCollider is a domain-specific audio programming language originally created by James McCartney and open sourced in 2002 [39]; SCMIR uses audio feature extraction algorithms available within the SuperCollider audio synthesis server, batch processing over a set of audio files in non-realtime mode. Calculation proceeds as fast as possible, typically a small fraction of the playtime of any given audio file; feature analysis of the corpus here took 56 min. Each independent feature was normalized according to interquartile range normalization to minimize the effect of outliers (subtract the median and divide by the range between the 25 and 75% percentiles); the normalization range for a given feature was calculated first across all audio files before application to that feature for each individual file with respect to the global norm. In the last column, table 3 gives the specific SuperCollider ‘UGens’ (signal processing modules) used in extraction; their SuperCollider help files give more detail on the algorithm, and all code is open source. More detail on machine listening processes in SuperCollider is also available in a chapter of *The SuperCollider Book* [40], and further technicalities of musical audio feature extraction are discussed in MIR textbooks [3,22].

**Table 3.** Timbral-rhythmic audio features extracted.

feature number	correlation rank	audio feature description	SuperCollider analyser
0	1	perceptual loudness	Loudness
1	5	Sethares sensory dissonance algorithm [36]	SensoryDissonance
2−4	8, 3, 2	low, mid, high energy	BLowPass4, BBandPass, BHiPass4
5−16	12, 16, 23, 17, 22, 18, 20, 24, 21, 26, 19, 27	first 12 Mel frequency cepstral coefficients (MFCCs)	MFCC
17	9	spectral centroid	SpecCentroid
18	10	50% spectral percentile	SpecPcile
19	11	90% spectral percentile	SpecPcile
20	13	spectral flatness	SpecFlatness
21	15	spectral entropy	SpectralEntropy
22	6	transient energy via Daubechies wavelet transform [37]	WT_Transient
23	7	Root Mean Square (RMS) energy of the harmonic part [38]	RunningSum.rms, MedianSeparation
24	4	RMS energy of the percussive part	RunningSum.rms, MedianSeparation
25−27	14, 25, 28	three onset statistics of the percussive part: in the previous two seconds, the density (raw count) of detected attacks, and the mean and standard deviation of the inter-onset intervals between successive attacks detected	OnsetStatistics, Onsets, MedianSeparation
28	29	metricity of the percussive part (consistency of larger energy beat histogram entries with the strongest such entry, according to whether the tempi correspond to a simple integer ratio, that is, clearly related metrical levels)	BeatStatistics, MedianSeparation
29	30	tempo	BeatTrack

Harmony was handled separately by the use of the open-source Chordino chord detection algorithm from the Vamp plugins^3^ [41], suitable for batch running from the command line through the Sonic Annotator software.^4^ Chordino provides the ability to extract 10 different chord types (discussed further in section 5 below), providing better granularity than the major–minor only chord extraction provided in the MIR libraries madmom^5^ [42] or Essentia [43].^6^ Chordino has been shown to be competitive in the most recent Music Information Retrieval EXchange (MIREX 2019) competition where more than one algorithm was compared,^7^ and has been available for around a decade as open-source software entered multiple times into earlier MIREX competitions [44]. In qualitative checks compared with human annotation, it was sufficiently reliable to use for this study, even if chord timings or repeats were not always entirely accurate (the analysis below rests more on qualities of chord present rather than fully accurate timings); it was also used for a previous preliminary examination specifically of the music of Radiohead, giving a continuity to the comparison herein [31].

Output from Chordino was post-processed with custom SuperCollider code to parse assigned chords and carry out local key detection sufficient to interpret chords with respect to harmonic function. For the latter routine, to assign a local key to every given chord, five chords were taken immediately preceding and following that chord, whenever available within a ±20 s time window, and a histogram over pitch classes formed from all pitches in those surrounding chords; this histogram was then cross-correlated with Krumhansl–Kessler profiles [45, p. 51] to find a best matching key profile across the major and minor keys. Harmonic function could then be assigned to consecutive chords in the same key, allowing the transposition-invariant and key-contextual labelling of chord transitions.

**Figure 1.**
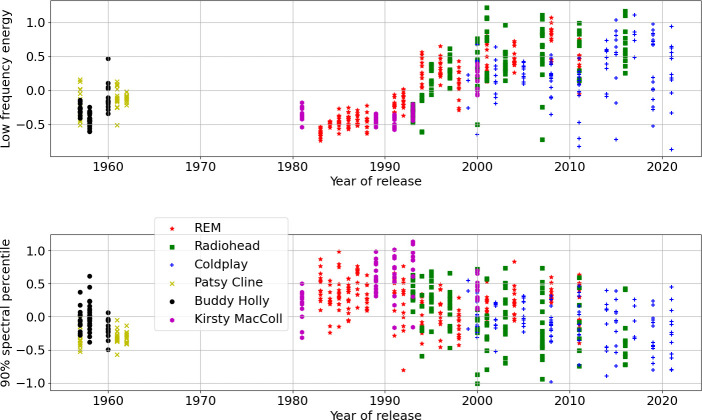
Comparative plot of the low-frequency energy and approximately 90% spectral percentile features by artist and year. Each plotted point is one song from the corpus.

As an example of insight from basic feature data, figure 1 plots the low-frequency energy (frequencies under 400 Hz) and the 90% spectral percentile against year of release. Coldplay’s music shows the widest range of bass volumes, and the overall trend of increased bass fits the picture offered in other research [11]. The fall in spectral percentile directly relates to the rise of bass volume, since more energy at lower frequencies in the spectrum will drop the frequency at which 90% of energy is achieved (energy in the mid-frequencies does not shift higher to compensate; we just have more prominent bass).

**Figure 2.**
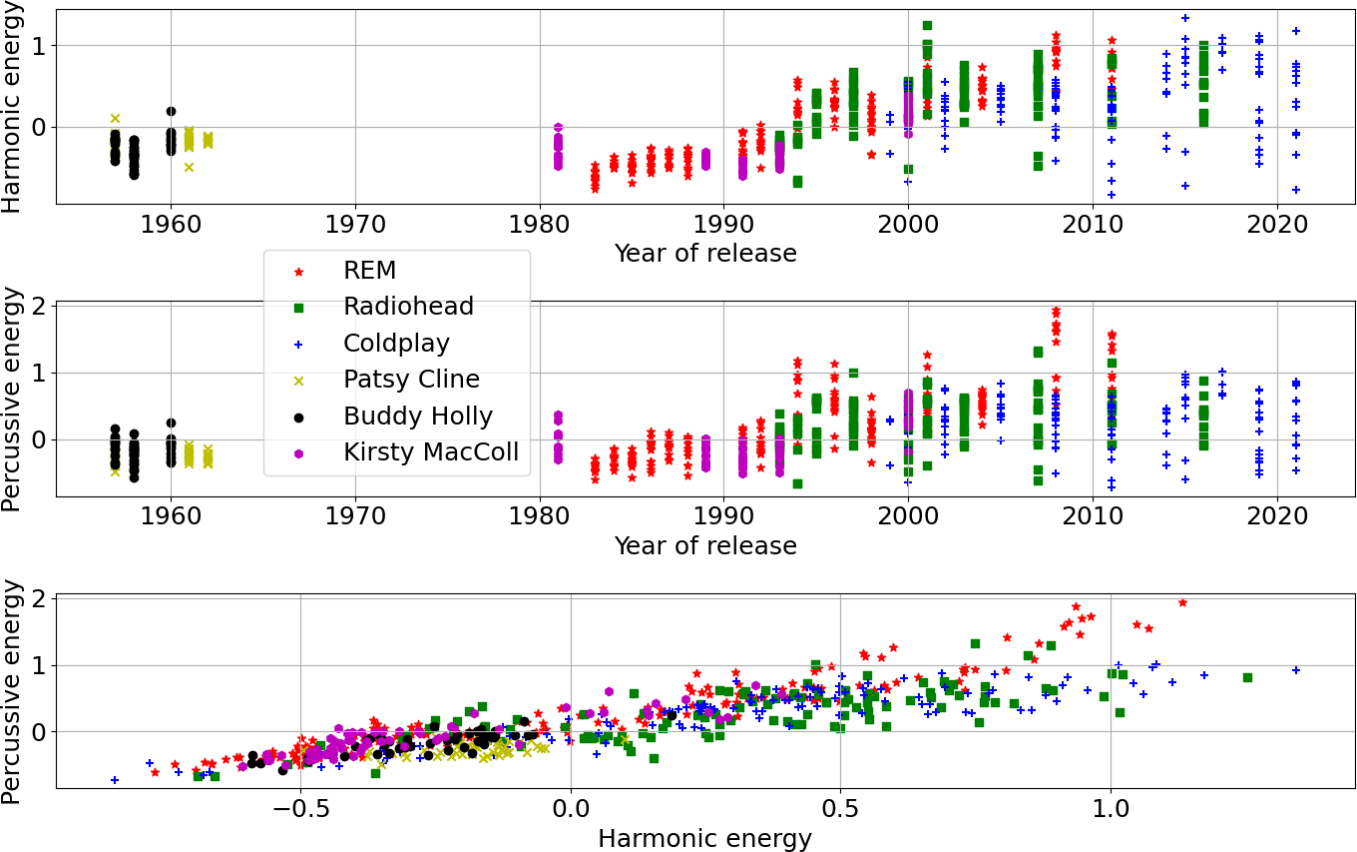
Three scatter plots showing the interactions of year of release, and the RMS energy of the harmonic and the percussive parts of song signals (one point per song in the corpus, colour-coded and marked following the convention established in figure 1).

Figure 2 presents a comparison of the harmonic and the percussive energy within songs, for the artists over time; the three linked plots show each of the interactions of two out of three dimensions between the release year and the two audio features (harmonic/percussive). Both the harmonic and percussive energy rise in keeping with the arrival of the Loudness War (peaking around 2007 according to Deruty & Pachet [13]); here, things never really recover to pre-1994 levels once there is a bump up, although post-2010 percussive energy does drop back a bit. The artists do not only release loud songs, and actually show a wider range of variation in energy levels between songs within given release years. Furthermore, the plot of harmonic versus percussive energy is quite a good discriminator of the three control artists from the louder excesses of the three alternative rock principals, even if the variety of signal levels in song recordings of R.E.M., Radiohead and Coldplay leads to some overlaps in the lower left that would not allow overall separability. The alternative rock acts thereby never entirely embrace aggressively loud volume songs, but stay for plenty of song recordings in check with earlier eras of recorded sound.

**Figure 3.**
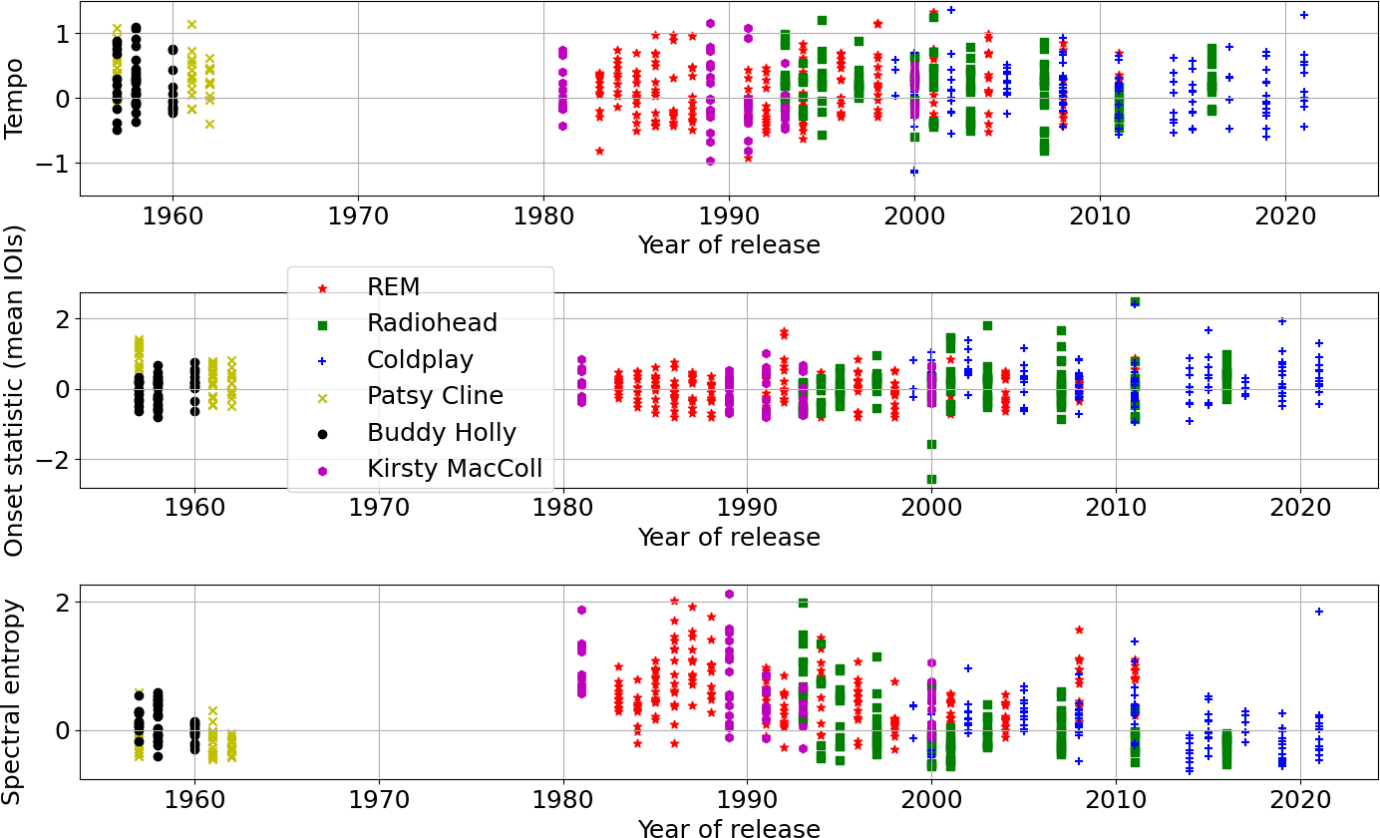
Scatter plots of tempo, the second onset statistic and the spectral entropy, chosen for minimal correlation scores.

Correlations between features led to feature versus feature plots often clustering in smaller volumes. Examining the (Pearson) correlation matrix^8^ for the set of features in table 3, the absolute magnitude of the matrix entries was taken and summed across rows (symmetrically by columns) giving the average absolute correlation of each feature with all others. Ordering this vector revealed the features from least to most correlated (the ranks are noted in the second column of table 3). Because timbral-rhythmic features are often connected to time domain signal strength through the measurement of time-varying spectral energy, it is unsurprising that the perceptual loudness bears the strongest relationship to the other timbral-rhythmic features here; conversely, the tempo and the metricity were the least correlated features overall. Figure 3 compares year of release against the tempo, the second onset statistic (mean Inter-Onset Interval (IOI)) and the spectral entropy, chosen as the least correlated triplet of features. The 1980s releases of R.E.M. and Kirsty MacColl show a higher spectral entropy, perhaps owing to faster-changing percussion from the 1980s production trend of gating drums, without any confound caused by the increase in later decades in bass and overall volume in mastering. The greater spectral entropy of Radiohead in the 1990s is attributable to their more distorted guitar-led rock sound at that point, before the increased turn to electronics and other expanded instrumentation with the album *Kid A* (2000); this echoes findings in an earlier research report specifically investigating 163 Radiohead songs [31]; there is a slight upwards return peaking at *In Rainbows* (2007) before the later and mellower last two albums. R.E.M. meanwhile return to a more raucous sound with their final two albums, belying any naive assumptions about rock stars mellowing with age. The tempi and the mean IOIs do not show any obvious large changes over time; there are neither particularly obvious year-by-year songwriting and production trends, or age-related effects such as slowing tempi over time. However, some songs deliberately investigate faster rushes of events which can trigger a flurry of detected onsets in the absence of conventional percussion (*Motion Picture Soundtrack* (2000) by Radiohead is one of the outliers on the mean IOIs plot at the bottom, Coldplay’s *Daddy* (2019) and Radiohead’s *Codex* (2011) are rather slow dirges). However, these features are not obviously discriminative of the artists, even if there are some variations in the range used per feature for artists; most songs cluster around the 0 mean of the feature, and plotting features against each other does not reveal any obvious separability of artist categories.

**Figure 4.**
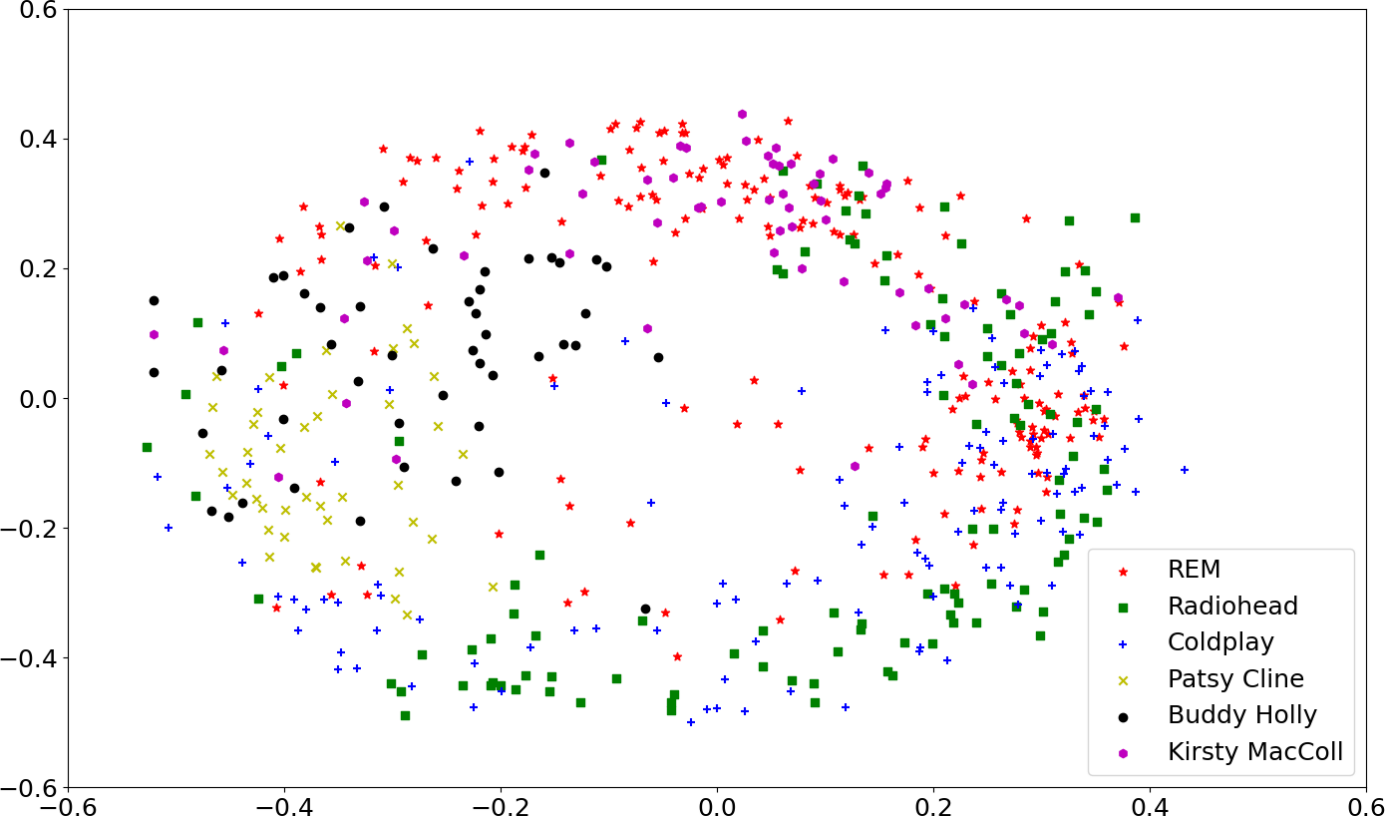
MDS plot reducing from the 30 feature space to a two-dimensional space, whose axes have no direct interpretation as a linear combination of the 30 features, but seek to preserve distances from the original space. Each point is one song, colour-coded for the six artists as commonly used throughout the figures in this article.

Figure 4 presents a multidimensional scaling (MDS) dimensionality reduction (sklearn’s manifold.MDS^9^) from the 30 feature space (one mean vector per song) to a two-dimensional embedding. All 573 songs are illustrated with the colour scheme and marker in common with other plots. The two-dimensional viewpoint does indicate visually some clustering of artists within particular quadrants; Patsy Cline and Buddy Holly only appear to the middle left, R.E.M. and Kirsty MacColl tend to be higher up the plot on the *y-*axis and Coldplay lower down, Radiohead more towards the right. However, there are also clear areas of overlap, especially on the right between R.E.M., Radiohead and Coldplay; there is no simple geometric solution for the separability of artists within this plot, and the audio features extracted are insufficient to give a clean separation of artists’ songs.

## 4. Similarity between artists and between albums

Artists were compared through the feature proximity of their songs with those of other artists. The most robust way to proceed was through the training of specific models for each given artist, which were then used to predict releases for that artist and the others in the study.

**Figure 5.**
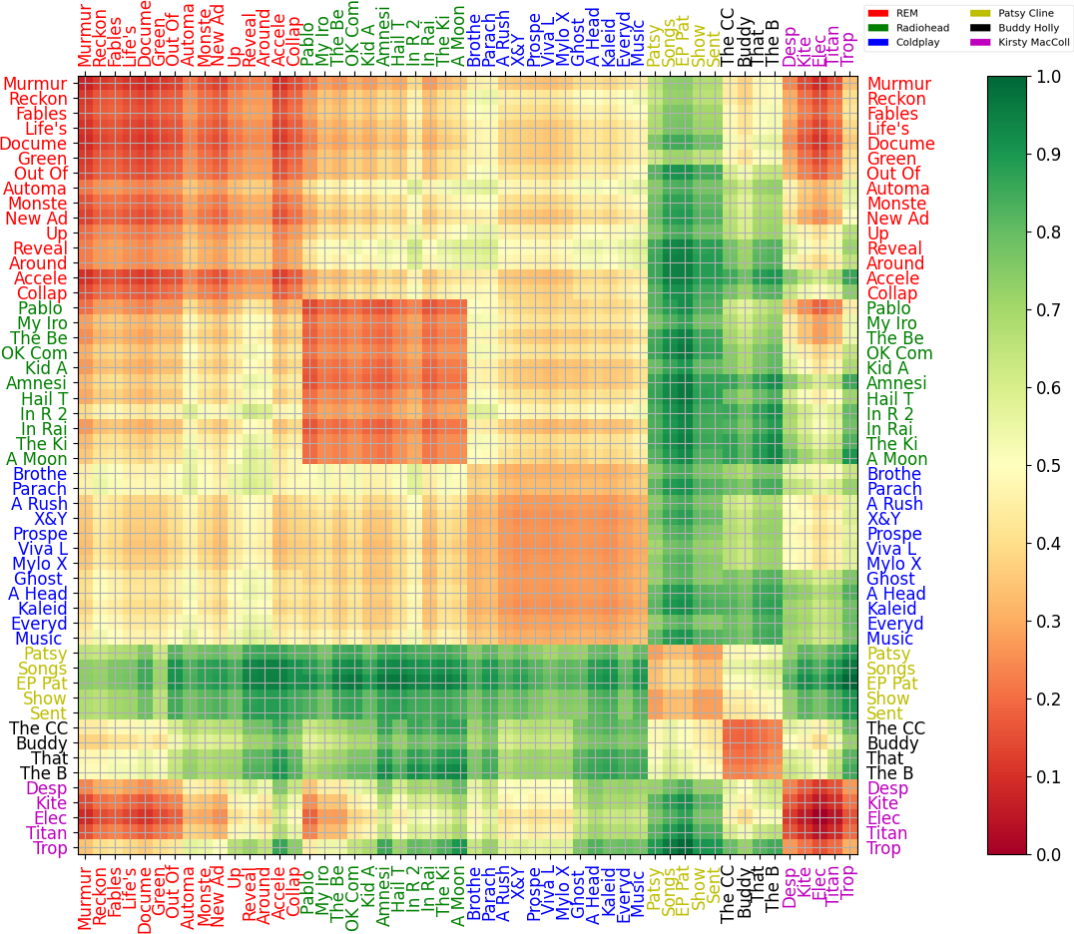
Dissmilarity matrix across albums derived from the symmetric log loss measured between variable order Markov models (one per artist), trained on kMeans discretisation of feature vectors (*k* = 30, max order 3). Smaller values correspond to greater prediction from a model, that is, greater similarity.

A similarity comparison was undertaken between the six artists by comparing the 52 releases of song collections (table 2). Two main methods were used; average feature vector comparison through Euclidean, Manhattan and cosine distance, and model construction after feature discretization. Since the latter technique led to more insight, it is described here in more detail; figure 5 plots the dissimilarity matrix across albums, where smaller values correspond to greater similarity (less error in model predictions). Each feature (already quartile normalized) was independently max–min normalized then discretized into 20 equally spaced bands by kMeans discovery of representative centroid vectors, and assignment of each feature vector to the closest centroid; feature vectors over a song thus led to an integer sequence per song. For a given artist, all artist song sequences became the training data for a variable-order Markov model (maximal order three). Having obtained six models over the artists, the 52 by 52 dissimilarity matrix was constructed by the symmetric log loss for each entry (i,j) as the sum of predicting album sequence data j from the artist model associated with album i and album sequence data i from the artist model associated with album j [46]. Results were much less reliable if 52 models, one per album, were constructed, so making models per artist but testing over albums was more robust. The matrix provides insight because it allows a comparison across all the releases in the present study, and helps to confirm that self similarity within an artists’ oeuvre is stronger than associations with other artists, but that there are still clear connections between artists.

The dissimilarity matrix is symmetric, with the checkerboard pattern on the diagram separating the set of albums for each artist (confirming local disparity at the transitions between artists). Some links between artists are apparent; Coldplay are more derivative of Radiohead from their second album (*A Rush Of Blood To The Head*) on, Kirsty MacColl’*Electric Landlady* has the closest links to Radiohead’s early albums (perhaps because of the common The Smiths link and the presence of Johnny Marr on Kirsty’s album), Buddy Holly and early R.E.M. have some overlap but Buddy and Patsy Cline are otherwise less connected to the other four artists. Coldplay have lighter links to other artists and within their own oeuvre; it might be that they have a less strong characterization by key influential albums, but slowly and consistently develop over time, as their career contrasts indie guitar and pop mainstream.

Table 4 presents a dissimilarity matrix over artists’ total releases, rather than grouped by albums, again with the symmetric log loss approach (the resulting matrix is therefore symmetric). Specific distances are presented in a tabular form; note that the values have been normalized by the maximum and minimum for ease of comparison, and smaller means closer (better predicted by the models of the two artists under comparison at a specific row–column intersection). The results are relatively sensible from a musicological standpoint; as a sanity check, all artists are best predicted by their own models (indicated along the main diagonal with italics). Although the proximity of R.E.M. and Kirsty MacColl is perhaps unexpected, they did start their careers within a few years of each other, and both are grounded in 1970s post-punk to 1980s indie culture. That Kirsty MacColl and Patsy Cline are further apart, given Kirsty’s various homages to country and their status as both female vocalists, is more surprising. Coldplay is most similar among the other artists represented here to Radiohead, Radiohead are closest to R.E.M., giving the expected influence chain of R.E.M.->Radiohead->Coldplay. Patsy Cline and Buddy Holly, separated from the others in an earlier era of popular music, do not well predict any of the later artists, demonstrating that there has been substantial change in music creation and production over time.

**Table 4.** Artist dissimilarity matrix; lower scores mean greater similarity, normalized within the 0–1 range. (The closest on each row aside from the obvious minimal self-dissimilarity along the main diagonal is indicated in bold; the diagonal is in italics. The only non-symmetric proximity is that R.E.M. and Kirsty MacColl are closest to each other, but Radiohead is closest to R.E.M. and Coldplay to Radiohead (thus, interestingly, in the order of chronological influence between the three alternative rock acts).

artist	R.E.M.	Radiohead	Coldplay	Patsy Cline	Buddy Holly	Kirsty MacColl
R.E.M.	*0.098*	0.274	0.325	0.905	0.621	**0.239**
Radiohead	**0.274**	*0.103*	0.29	0.993	0.874	0.5
Coldplay	0.325	**0.29**	*0.185*	0.927	0.887	0.541
Patsy Cline	0.905	0.993	0.927	*0.255*	**0.511**	1.0
Buddy Holly	0.621	0.874	0.887	**0.511**	*0.172*	0.575
Kirsty MacColl	**0.239**	0.5	0.541	1.0	0.575	*0.0*

## 5. Comparison of harmony

**Figure 6.**
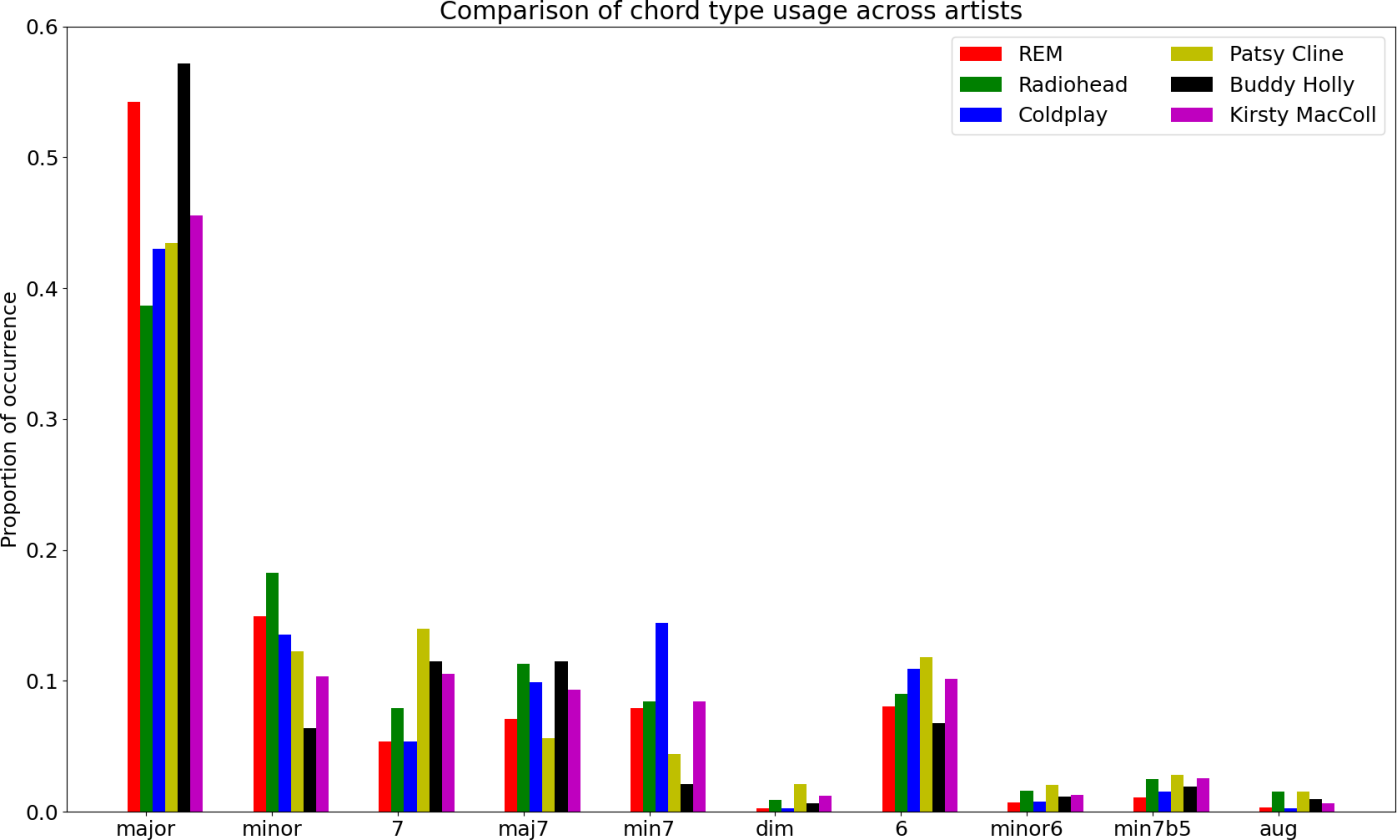
Relative occurrence of chord types for each artist, on a common bar chart (proportions are relative to total number of chords available in the data for each artist).

Figure 6 groups on a common bar chart the usage by the six music artists of 10 chords types extracted by the chord detection algorithm (as introduced in §3). Certain aspects of the data accord with common sense; Radiohead use the most minor chords of the six artists here, but Patsy Cline makes good use of dominant seventh and diminished harmonies (with jazz swing and doo-wop 50s harmonies amongst her song styles). Buddy Holly and R.E.M. are quite major in their chord outlook, and Coldplay’s compositions often involve minor seventh chords. Table 5 explores the top five functional harmonic transitions for each artist (where for example I means the tonic major, i the tonic minor, IV the subdominant major etc. [47]). Only Radiohead and Patsy Cline incorporate any minor chords in their top five transitions; Radiohead make frequent use of I->i progressions directly from major to minor, and iv->I (minor subdominant back to tonic) and Patsy Cline uses the well-known major to relative minor I->vi (which is the first step in the ‘50s’ progression used for doo-wop and much subsequent popular music).

**Table 5.** Top five chord transitions for each artist, as functional harmony after local key analysis. (The p columns p1 to p5 give the proportion of all chord transitions of the given type; the proportions drop off quite quickly (see also figure 7).)

artist	p1	transition1	p2	transition2	p3	transition3	p4	transition4	p5	transition5
R.E.M.	0.0646	IV ->I	0.0541	I ->IV	0.0257	I ->V	0.0231	V ->I	0.0199	V ->IV
Radiohead	0.0342	IV ->I	0.0318	I ->IV	0.0112	I ->i	0.011	IV maj7 ->I	0.0106	iv ->I
Coldplay	0.0559	IV ->I	0.0339	I ->IV	0.0304	I ->V	0.0219	V ->I	0.0216	IV maj7 ->I
Patsy Cline	0.0377	I ->IV	0.027	IV ->I	0.0246	V ->I	0.0205	V7 ->I	0.0135	I ->vi
Buddy Holly	0.1005	I ->IV	0.0823	IV ->I	0.0477	V ->I	0.0329	V7 ->I	0.0306	I ->V
Kirsty MacColl	0.0523	I ->IV	0.0466	IV ->I	0.0294	V ->I	0.0174	V7 ->I	0.0169	I ->IV maj7

**Figure 7.**
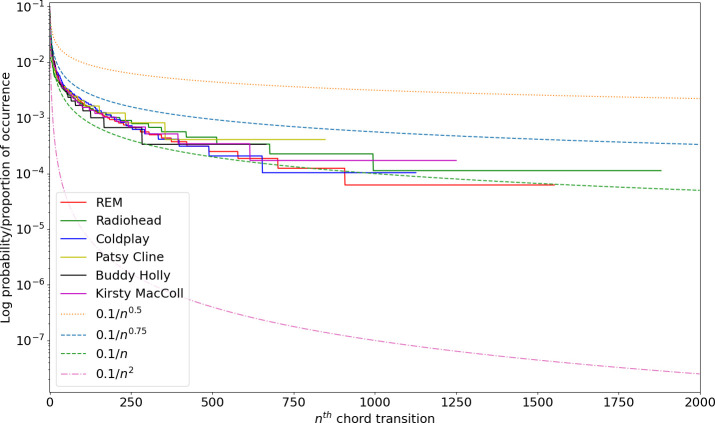
Relative occurrence of chord transitions for each artist, compared with $0.1/n^{k}$ curves for k=0.5,0.75,1,2, with a log on the *y*-axis for clarity.

Figure 7 plots the log probability of chord transitions explored by each artist from the most popular to the least used (that is, normalized by the total number of chord transitions for each artist; some artists use a smaller number of transitions than others; hence, the running out of the respective lines for some *n* on the *x-*axis). The fall-off is generally around a 0.1/n curve, mirroring previous studies of ‘Zipfian’ $1/n^{\alpha }$ rules for corpora usage in text and symbolic music [48,49]. While some artists have a smaller vocabulary of transitions explored in their music, the curves across artists generally remain within $0.1/n^{0.75}$ to 0.1/n; however, the curve for these popular music artists is further from a $1/n^{2}$ rule seen in the chord transitions of Bachian harmonic practice [50], and distinct from the $1/n^{1.6}$ to $1/n^{2.4}$ often observed in corpus studies of communicative language [51].

**Figure 8.**
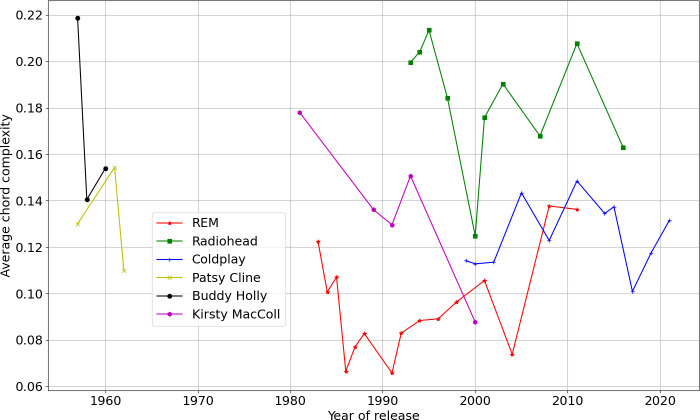
A measure of chord complexity is plotted for each artist taking the average complexity of harmony for their songs in a given release year.

A measure of chord complexity can be defined based on an artist’s usage of the more complex harmonic possibilities amongst the 10 chord types treated here. For this purpose, the chord types major seventh, diminished, minor sixth, minor seventh b5 and augmented (*x*-axis indices 3, 5, 7, 8, 9 in figure 6) are taken as being ‘complex’. The chord complexity measure is the ratio of the number of ‘complex’ chords to all chords appearing, that is, the proportion of more complex harmony used. Figure 8 plots this chord complexity measure by year, taking the mean of the chord complexity for an artist’s songs from a given release year. The trend for Kirsty MacColl towards a simpler harmonic language for her final Latin album *Tropical Brainstorm* (2000) is clear; Patsy Cline also seems to simplify for early 1960s popular music. Interestingly, the timbral studio experimentation for Radiohead which peaked with *Kid A* (2000) and *Amnesiac* (2001) is reflected in a simpler harmonic language for them at this time in compensation to the listener, despite an overall greater level of harmonic adventure in their career relative to other artists here. R.E.M. are not the greatest explorers of harmony, and Coldplay are intermediate between R.E.M. and Radiohead.

## 6. Timbral-rhythmic and harmonic diversity of artist output

Diversity measures of audio features appearing in any given song, or any collection of songs in a particular year, or for any artist overall, can be calculated, given a discretization of the feature data. Two diversity measures were investigated here, Shannon entropy and Simpson’s *D* [52]; since Shannon entropy gave very similar results to Simpson’s *D*, only the latter is reported.

**Figure 9.**
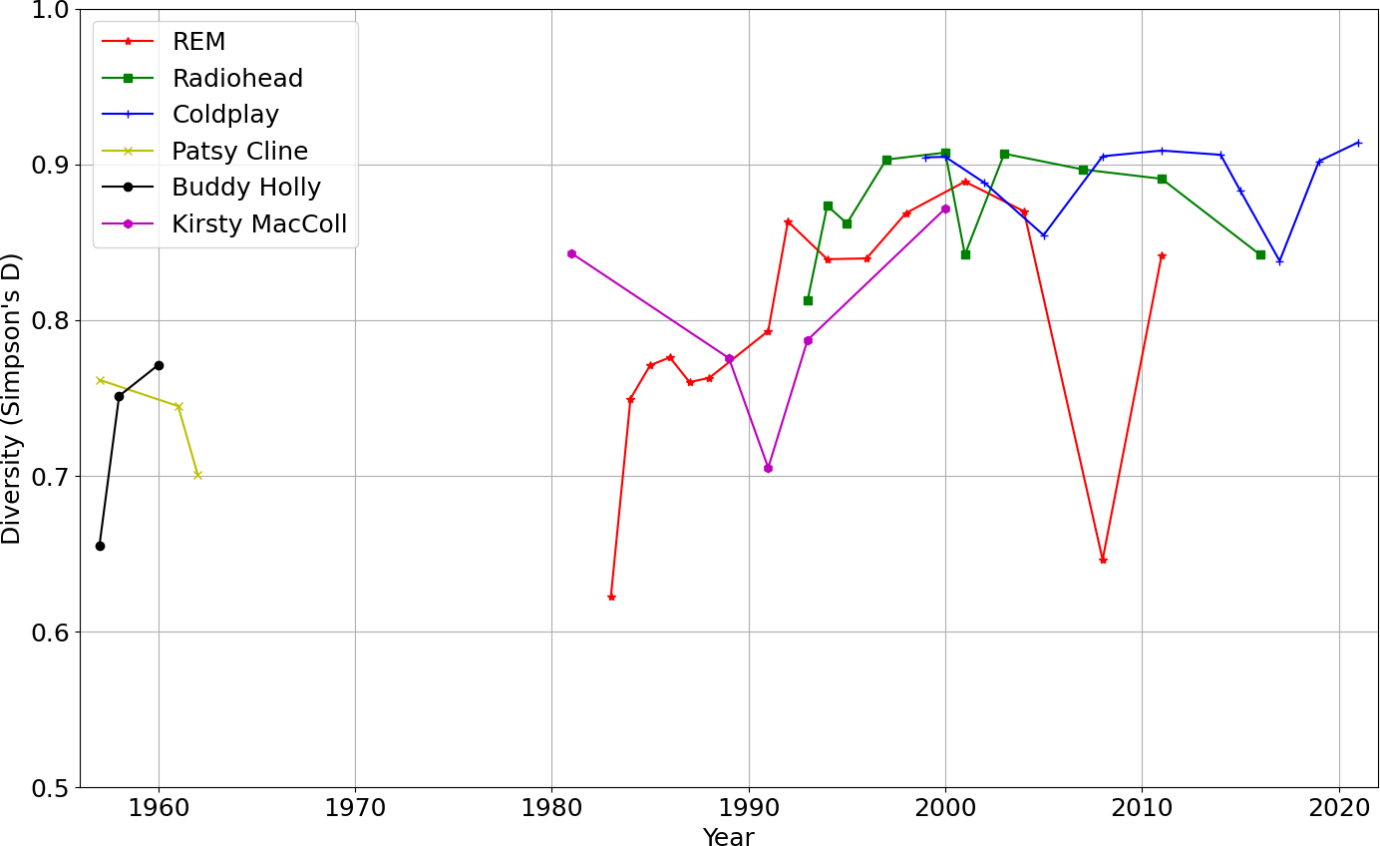
Diversity per year (Simpson's *D*) with respect to a discretisation of timbral-rhythmic audio features.

**Figure 10.**
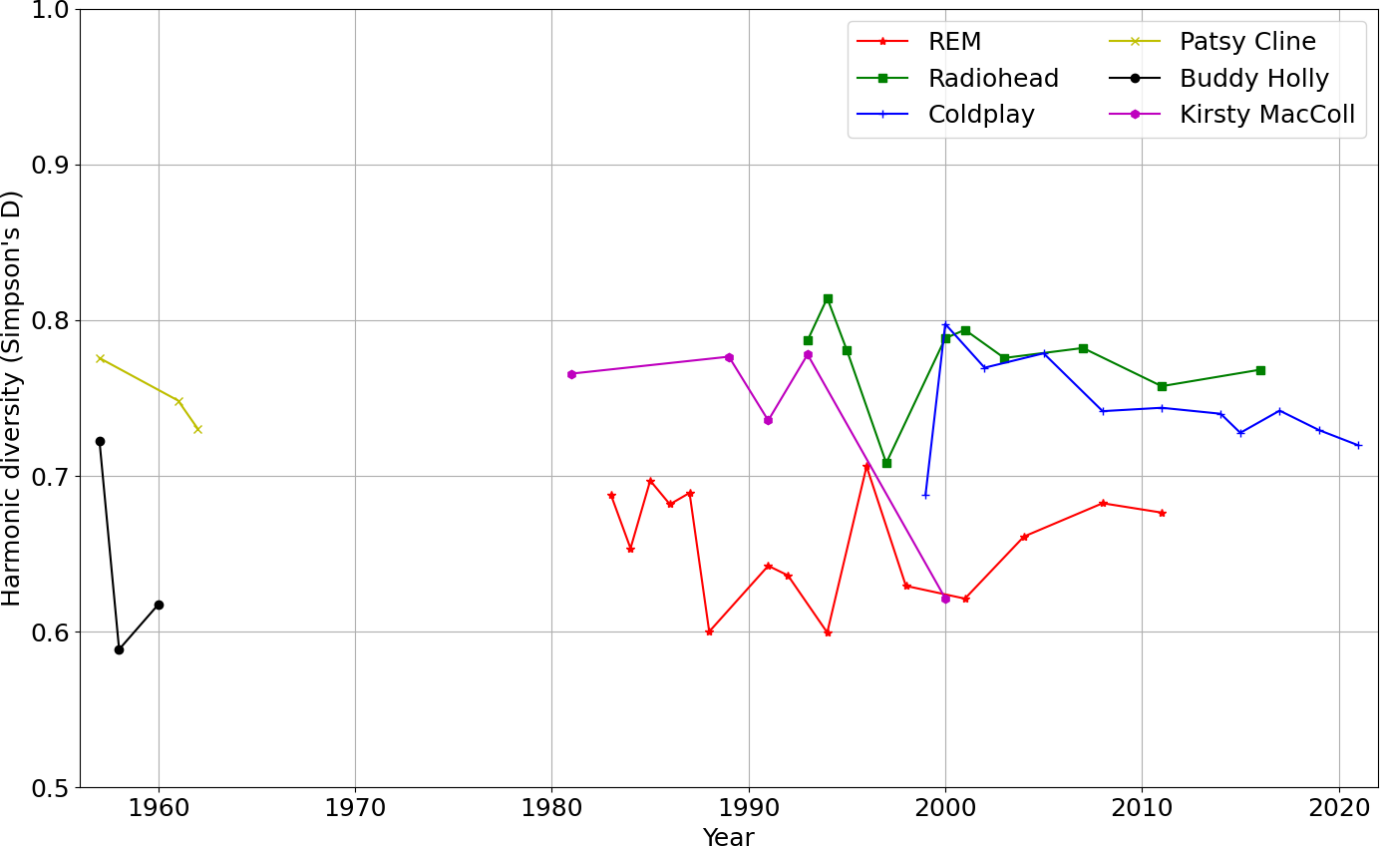
Harmonic diversity per year (Simpson’s *D*).

Figure 9 treats year-by-year audio feature diversity according to the Simpson’s *D* measure for each of the six artists, with respect to the 20 centroid kMeans model used for similarity matrix Markov modelling in §4. Varying the number of centroids (for example to 100) made little difference to the shape of the plot. The earlier artists Patsy Cline and Buddy Holly, and much R.E.M., is less diverse in the discretized space of timbral-rhythmic feature vector representatives. Radiohead and Coldplay fluctuate, but are consistently more wide ranging. Figure 10 applies the Simpson’s *D* measure for each of the six artists with respect to harmonic materials (chord type histograms across the 10 categories as also used in §5). Radiohead are the most consistently diverse in their harmonic language. It is clear again (see §5) that Kirsty MacColl’s final more Latin album *Tropical Brainstorm* (2000) simplifies the harmonic language, while increasing timbral-rhythmic variety; this trade-off of harmonic and timbral-rhythmic complexity, as also seen with Radiohead circa 2000−2001, is a recurring feature worth further future investigation.

## 7. Discussion

Whether there are prominent music compositional changes in a band’s output over time has been investigated in this article. The data as analysed accord mostly with expectations ahead of the study even if a few musicological surprises were found. Although the robustness of audio feature analysis may be questioned, analysis through audio chord detection was quite effective. I revisit the questions posed at the start of the article with a few immediate examples:

(i) *are there changes across an artist’s career observable from audio recordings through automated feature extraction? Can particular critical inflection points be observed, or indicators of early or late work?* It was certainly possible to see changes in the timbral-rhythmic and harmonic content of recorded releases varying over time for a given band. Kirsty’s MacColl’s last album is a strong case in point, where harmonic complexity is reduced to accommodate increased timbral-rhythmic emphasis in Latin rhythms and texture;
(ii) * relative to comparator artists, what diversity of recorded output is achieved by a given band with respect to particular musical attributes such as complexity of rhythm, harmonic language, or size of timbral space explored?* Section 6 examined this, finding for example that Radiohead were consistently more diverse than Coldplay harmonically, but not necessarily timbrally; and
(iii) *are there any commonalities of career, such as a general slowing of tempi or preference for less high-frequency content in textures (for example, less distortion in rock) as musicians age?* R.E.M.’s late albums actually show a return to a heavier rock sound (clear in the upturn in spectral entropy in figure 3); tempi do not necessarily reduce for later albums.

Where ‘rock n roll’ in the 1950s may have been closely associated with the rise of the independent social status of the teenager, and a certain ageism continues to pervade pop, the longevity of career of rock musicians since the 1960s, and the longevity of their fans, shows that a longer view of artist careers is warranted [53]. Ageing singers may see changes in their vocal physiology, but this is only more acute by their 70s and ameliorated by practise [54]. Studies of ageing effects have the confound that no music artist has the same length career, nor the same social and musical circumstances; nobody makes the same choices. From the data here, variation in timbral, rhythmic and harmonic exploration can occur at different stages for different artists, and is not a simple function of age. Any cliched correlation of older musicians’ work to a musical preference for slower, less adventurous materials is not borne out by this study; when Kirsty MacColl simplifies her harmonies for her final album, it is because her engagement with Latin music has ramped up rhythmic and timbral possibilities, and there is no basis to predict that should she have lived longer, further albums would have continued to follow such a trend; witness the fluctuations over a career with more albums such as that of Radiohead.

The facility of the choice of control artists in this study was corroborated by the separate status of the 1950s artists Buddy Holly and Patsy Cline, whose signature recorded sound was more distant than the studio preferences of later decades (when multi-tracking became so much more standard and allowed much longer and more intricately edited recording sessions than the one take in a reverberant environment mode of much 1950s production). While they both had careers cut short, the recording industry at the time often pushed fast album releases to maximize revenue from ephemeral success; Buddy Holly’s complete output is from within a 2 year period of recording, but still shows evolution; Patsy Cline investigates many styles outside of any expected central country sound (for example, ‘*Too Many Secrets*’ (1957) is a swing number). The slower-paced releases of Kirsty MacColl (who had to work against a backdrop of contractual problems with her first label), overlapping with the three main rock artists considered here, show a similar concern to seek out new compositional possibility, and perhaps this restless movement in reaction to previous recordings is a primary artistic tendency. A larger scale study would be needed to validate the degree to which all bands keep changing and whether any stagnate much more. For instance, the UK music press had a stereotypical view of the band ironically called Status Quo as unchanging three chord dinosaurs, but whose 1970s output has been argued to be much more innovative [55].

A criticism of multiple reviewers of this work was the specificity of the artists being examined, and concerns over the generalizability of results. From a musicological perspective, the familiarity of the analyst with the music being studied precludes widening the set of materials too far straight away, and it gives a greater sense of confidence in examining results to know the music well. Nonetheless, the techniques explored here are applicable to other music artists outside this admittedly culturally constrained starting point. Female artists, female-fronted and all-female bands would be an interesting comparative study to that presented here, for example, looking at such artists as The Slits, The Bangles, PJ Harvey, The Breeders, or Björk. The present study might be embedded in a larger network of recorded music, exploring the relationship of a given artist to wider cultural trends. Computationally tractable year-by-year aspects of the music data may be expanded to include sentiment analysis of lyrics, or preprocessing with source separation and analysis between extracted stems (such as all bass parts, or all vocals). Computational analysis of influence may also be investigated, although is not necessarily as simple as chronological precedence. Although a band active in earlier years may seem to have an innate advantage in terms of influence, in practice, there can be a back-and-forth interaction of any bands contemporaneously releasing recordings. The cross-influence of The Beatles and The Beach Boys is a well-known example, especially around the time of *Pet Sounds* (1966) and *Sgt. Pepper’s Lonely Hearts Club Band* (1967). Radiohead were certainly influenced by R.E.M., but R.E.M. deliberately selected Radiohead as their support act for one tour, and Michael Stipe was so enamoured by *OK Computer* that he sang along from the side of the stage at a subsequent Radiohead festival performance before joining them to sing *Lucky* [28]. Audio content analysis is the primary investigative method of this research; future work might also analyse additional meta-data concerning the artists, such as journalism, online folksonomies, and a wider network of cultural reference points including country-specific comparison of the United States and UK music scenes and industries.

## 8. Conclusion

A comparative approach to the computational analysis of audio was undertaken, dissecting major recordings from six artists’ careers from the United States and the UK. Although two of the control cases had careers that were cut short by 1959 and 1963, Kirsty MacColl was actively releasing over two decades or so up to the year 2000, showing a similar fluctuation of timbral-rhythmic and harmonic complexity as the main bands in this study. The three bands all vary in their musical pre-occupations with different albums within the core period 1983−2021; R.E.M. are overall the least radical in the musical parameters extracted here. Coldplay shows a downwards trend in their harmonic daring as they increasingly become part of the pop mainstream, while exploring new timbral presentations of their music; Radiohead maintain a high diversity of harmonic language while their timbral-rhythmic variety also falls off a little with later albums. Overall, there were no obvious observed ageing effects. A much larger scale study may proceed from the seeds planted here to investigate these questions with larger corpora, though may lose some of the particular connection to specific artists. There is certainly a role for MIR audio content methods within computational musicology, in future analysis of recorded music careers.

## Data Availability

Recording Artist Career Comparison using SuperCollider and Python: a repository of all code used for the study ‘Recording Artist Career Comparison Through Audio Content Analysis’ for the corpus analysis with the SCMIR library in SuperCollider and further analysis and figure plotting in Python is available online [56].

## Footnotes

^1^
Catalogues of recordings by New Order or The Smiths would have to contend with many more non-album track singles releases.

^2^

https://github.com/sicklincoln/SCMIR. https://github.com/sicklincoln/SCMIR

^3^
vamp:nnls-chroma:chordino:simplechord.

^4^

https://vamp-plugins.org/sonic-annotator/

^5^

https://madmom.readthedocs.io/en/v0.16/modules/features/chords.html

^6^

https://essentia.upf.edu/reference/std_ChordsDetection.html

^7^

https://www.music-ir.org/mirex/wiki/2019:Audio_Chord_Estimation_Results

^8^
The full matrix and analysis code is supplied in the source code accompanying this article in the SuperCollider code file correlationmatrix.scd.

^9^

 https://scikit-learn.org/0.16/modules/generated/sklearn.manifold.MDS.html
